# Low-Dose Formaldehyde Delays DNA Damage Recognition and DNA Excision Repair in Human Cells

**DOI:** 10.1371/journal.pone.0094149

**Published:** 2014-04-10

**Authors:** Andreas Luch, Flurina C. Clement Frey, Regula Meier, Jia Fei, Hanspeter Naegeli

**Affiliations:** 1 Department of Product Safety, German Federal Institute for Risk Assessment (BfR), Berlin, Germany; 2 Institute of Pharmacology and Toxicology, University of Zürich-Vetsuisse, Zürich, Switzerland; University of Pittsburgh, United States of America

## Abstract

**Objective:**

Formaldehyde is still widely employed as a universal crosslinking agent, preservative and disinfectant, despite its proven carcinogenicity in occupationally exposed workers. Therefore, it is of paramount importance to understand the possible impact of low-dose formaldehyde exposures in the general population. Due to the concomitant occurrence of multiple indoor and outdoor toxicants, we tested how formaldehyde, at micromolar concentrations, interferes with general DNA damage recognition and excision processes that remove some of the most frequently inflicted DNA lesions.

**Methodology/Principal Findings:**

The overall mobility of the DNA damage sensors UV-DDB (ultraviolet-damaged DNA-binding) and XPC (xeroderma pigmentosum group C) was analyzed by assessing real-time protein dynamics in the nucleus of cultured human cells exposed to non-cytotoxic (<100 μM) formaldehyde concentrations. The DNA lesion-specific recruitment of these damage sensors was tested by monitoring their accumulation at local irradiation spots. DNA repair activity was determined in host-cell reactivation assays and, more directly, by measuring the excision of DNA lesions from chromosomes. Taken together, these assays demonstrated that formaldehyde obstructs the rapid nuclear trafficking of DNA damage sensors and, consequently, slows down their relocation to DNA damage sites thus delaying the excision repair of target lesions. A concentration-dependent effect relationship established a threshold concentration of as low as 25 micromolar for the inhibition of DNA excision repair.

**Conclusions/Significance:**

A main implication of the retarded repair activity is that low-dose formaldehyde may exert an adjuvant role in carcinogenesis by impeding the excision of multiple mutagenic base lesions. In view of this generally disruptive effect on DNA repair, we propose that formaldehyde exposures in the general population should be further decreased to help reducing cancer risks.

## Introduction

In aging populations, the incidence of chemoresistant malignancies continues to rise. For example, the ultraviolet (UV) radiation of sunlight is a primary risk factor for skin cancer [1]. As a major interface separating the body from the environment, skin cells provide an effective barrier against physical and chemical insults. Besides the photoprotectant melanin, a dedicated nucleotide excision repair (NER) activity mitigates the carcinogenicity of sunlight by excising UV-induced photoproducts from DNA before they are converted to genetic mutations [2], [3]. Another defense line, known as base excision repair (BER), removes concurrent oxidative base lesions [3]–[5]. However, the skin and other tissues frequently come in contact with chemicals that react with cellular components like chromatin in the close vicinity of DNA, thus potentially interfering with DNA-repairing enzymes. Formaldehyde is a compound of particular concern because of its ubiquitous distribution, widespread human exposure and verified human carcinogenicity. This reactive aldehyde appears in automotive emissions or tobacco smoke and is added to many industrial and medicinal products. Formaldehyde-containing resins are used in the manufacture of plywood, paper, textile fibers, plastics, paints, lubricants and dyes. Formaldehyde is also employed in furniture, upholstery, carpeting, drapery and other household products [6]–[9]. Cosmetics are another important source as they are often preserved with formaldehyde donors [10].

At high doses, formaldehyde generates DNA-protein crosslinks (DPXs) [11], [12] and induces nasal carcinomas in rodents [13]. In view of the documented risk of nasopharyngeal, sinonasal, lymphatic and hematopoietic cancer in occupationally exposed workers [14]–[16], it has been categorized as a human carcinogen [17], [18]. This current classification does not consider formaldehyde as a possible risk factor for cutaneous cancer, although very substantial levels of this compound (65% of the dose) are found in the skin after topical application to experimental animals [19]. This pronounced ability of formaldehyde to penetrate the skin raises the concern that cutaneous cancer may represent another adverse endpoint. In an animal model of carcinogenesis, formaldehyde exhibits no tumorigenicity by dermal exposure on its own but nevertheless displays a deleterious effect by dramatically reducing the latency time of carcinogen-initiated skin tumors [20]. Such an adjuvant role during the carcinogenesis process, detected in animal experiments, is also supported by the observation that morticians, who used formaldehyde as an embalming fluid, display an elevated mortality due to skin cancer [21].

Previous studies suggested an interference of formaldehyde with the proper processing of various forms of DNA damage [22], [23]. In the case of global-genome NER activity, the detection of UV lesions depends on a specific accessory complex, known as UV-DDB (for UV-damaged DNA-binding), which consists of a damage sensor (DDB2) and a regulatory subunit (DDB1) [24]. To explore the basis of the observed adjuvant effect in skin carcinogenesis, we tested how formaldehyde, at non-cytotoxic concentrations, influences the activity of this critical DNA damage recognition complex. We thereby identified a novel mechanism by which NER activity is inhibited in formaldehyde-exposed cells.

## Materials and Methods

### Expression constructs

The XPC complementary DNA [25] was cloned into pGFP-N3 (Clontech) using the restriction enzymes *Xma*I and *Kpn*I. The DDB2 complementary DNA was transferred from pGFP-DDB2-C1 (Dr. S. Linn, University of California, Berkeley, California) to pmRFP1-C3 using its *BamH*I sites. The pGFP-OGG1-C1 vector was obtained from Dr. P. Radicella, Institut de Radiologie Cellulaire et Moleculaire, Fontenay-aux-Roses, France, and the pGFP-APE1-C1 vector from Dr. H. Lans, Erasmus University Rotterdam, The Netherlands.

### Cell culture

All culture media and supplements were from Invitrogen. Simian virus 40-transformed fibroblasts (GM00637) were obtained from the Coriell Institute for Medical Research (Camden, NJ). They were grown in a humidified incubator at 37°C and 5% CO_2_ using Dulbecco's modified Eagle's medium (DMEM) supplemented with 10% heat-inactivated fetal calf serum, 100 units/ml penicillin G and 100 μg/ml streptomycin.

### Transfections

Fibroblasts (∼500,000) were seeded into 6-well plates. After 24 h, at a confluence of 80–85%, the cells were transfected with 1 μg expression vector using 4 μl FuGENE HD reagent (Roche). Following a 4-h incubation, the transfection mixture was replaced by complete medium and the cells were incubated for another 18 h at 37°C.

### Exposures

Solutions of 37% aqueous formaldehyde (Fluka) or acetaldehyde (Sigma) were each serially diluted in complete medium. All formaldehyde and acetaldehyde stocks were made fresh and maintained on ice to prevent evaporation. Cisplatin (*cis*-diamineplatinum-II, Sigma) was dissolved in dimethyl sulfoxide. After transfections, the cells were incubated with fresh complete medium containing again the indicated concentrations of formaldehyde or acetaldehyde. For irradiation, the medium was temporarily removed and the cells were rinsed with phosphate-buffered saline (PBS) before exposure to UV-C light from a germicidal lamp (257 nm wavelength). This wavelength results in the formation of cyclobutane pyrimidine dimers and (6–4) pyrimidine-pyrimidone photoproducts, which constitute the most prevalent forms of DNA damage induced by solar light [26].

### Protein dynamics

**Figure 1:**
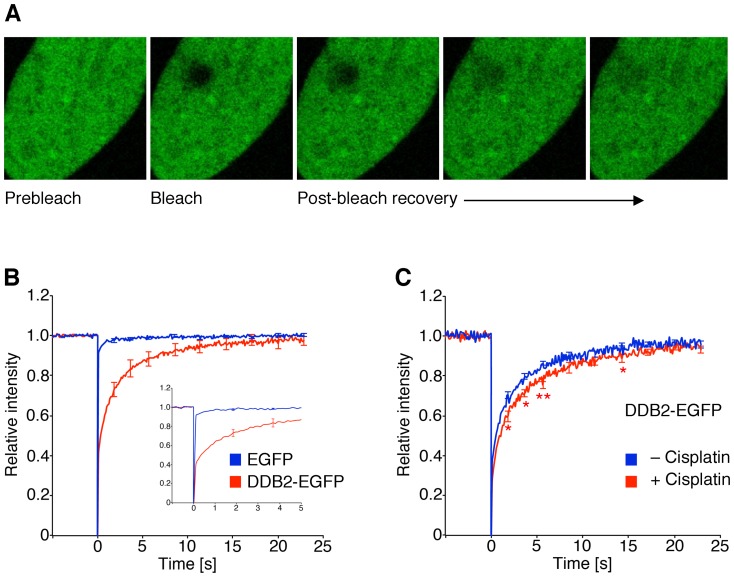
Analysis of repair protein dynamics in living cells. (**A**) A region of interest of ∼4 μm^2^ was photo-bleached and the fluorescence recovery within this area was monitored over a time frame of 23 seconds. Simultaneously, a reference area of the same size was monitored to correct for overall bleaching and the resulting data were normalized to the pre-bleach intensity. (**B**) Quantitative FRAP recordings determined in fibroblasts transfected with expression vectors coding for the DDB2-EGFP fusion or the EGFP moiety alone (N = 30; error bars, S.E.M.). (**C**) Recognition of cisplatin-DNA adducts by UV-DDB revealed by FRAP analyses. Human fibroblasts transfected with the DDB2-EGFP construct were pre-incubated with 5 μM cisplatin. The resulting FRAP curves were compared with those of untreated controls. Asterisks, statistically significant differences between cisplatin-treated cells and untreated controls (N = 50; *p<0.05; **p<0.01).

High-resolution fluorescence recovery after photobleaching (FRAP) analyses were carried out under a controlled environment at 37°C and with a CO_2_ supply of 5% using a Leica TCS SP5 confocal microscope equipped with an Ar^+^ laser (488 nm) and a 63× oil immersion lens (numerical aperture of 1.4) as illustrated in **Figure 1A**. A region of interest of ∼4 μm^2^ was photo-bleached for 2.3 s at 80% laser intensity. Fluorescence recovery within the region of interest was monitored 200 times using 115-ms intervals followed by 30 frames at 250 ms and 20 frames at 500 ms. Simultaneously, a reference area of the same size was monitored throughout all time points to correct for overall bleaching. Finally, the data were normalized to the pre-bleach intensity.

### Chromatin-binding assay

Salt extraction and micrococcal nuclease (MNase) digestion was used to analyze the binding of UV-DDB to chromatin [25]. After electrophoretic separation, the samples were transferred to a polyvinylidene (PVDF) membrane (BioRad) blocked by incubation for 2 h at room temperature with Tris-buffered saline containing 0.05% (v/v) Tween-20 and 5% (w/v) nonfat dry milk. Antibodies against the following proteins were used in Western blots: DDB2 (dilution 1∶50, ab51017, Abcam), GAPDH (1∶4′000, No. 4300, Ambion), H3 (1∶10′000, No. 07-690, Millipore). HRP-conjugated secondary antibodies were diluted 10,000-fold. Reactions were developed with SuperSignal West Pico (Pierce), recorded with a FUJI LAS-3000 imaging system and quantified using the Quantity One software (BioRad).

### Induction of UV foci

Human fibroblasts were grown on glass cover slips (20 mm diameter) and transfected with DDB2-EGFP or XPC-EGFP constructs. After 18-h incubations, the cell culture medium was removed and the cells were rinsed with PBS. UV foci were induced by irradiation through the 5-μm pores of polycarbonate filters (Millipore) using a UV-C source (257 nm, 150 J/m^2^). After irradiation, the filters were removed and the cells incubated for 15 min at 37°C in complete DMEM. Immunocytochemistry was carried out as described [25].

### Colony-forming assay

Fibroblasts were treated with formaldehyde as described above, UV-irradiated at increasing doses, seeded in different dilutions and incubated in cell culture medium for 7 days at 37°C to allow for colony formation. Colonies were stained with 0.5% (w/v) crystal violet in 80% ethanol and counted.

### DNA repair assays

The pGL3 and phRL-TK vectors were from Promega. The pGL3 vector was UV-irradiated (257 nm, 1000 J/m^2^) on ice in 10 mM Tris-HCl (pH 8) and 1 mM EDTA. Alternatively, pGL3 was incubated with 5.4 μM cisplatin for 24 h at 37°C. The modified plasmids were recovered by ethanol precipitation. To generate 8-oxo-2′-deoxyguanosine (8-oxo-dG) lesions, pGL3 DNA was mixed with 2 μM methylene blue and irradiated for 60 min on ice with visible light using a tungsten lamp (75 W) at a distance of 20 cm. Subsequently, the dye was extracted with 1% (w/v) sodium dodecyl sulfate and Tris-EDTA-saturated 1-butanol [27]. Human fibroblasts, grown to a confluence of 80% in 6-well plates, were transfected with 0.45 μg pGL3 DNA and 0.05 μg phRL-TK. After a 4-h incubation, the transfection reagent was replaced by complete medium. The cells were lysed after a further 18-h period using 0.5 ml Passive Lysis Buffer (Promega) according to the manufacturer's instructions. *Photinus* and *Renilla* luciferase activity was determined in a Dynex microtiter plate luminometer using the Dual-Luciferase Assay System (Promega). For the assessment of DNA repair activities, mean values were calculated from the ratios between *Photinus* and *Renilla* luciferase activity [28]. Antibodies against UV lesions (MBL International) were used in a slot-blot assay following the manufacturer's instructions.

### Statistical procedures

All results were analyzed with Prism 5 (GraphPad Software) using the Student's *t*-test for comparisons. A value of p<0.05 was considered statistically significant. The number of independently repeated experiments (N) is indicated in each figure legend.

## Results

### Analysis of UV-DDB dynamics in living cells

To test whether formaldehyde disturbs the cellular trafficking of the critical UV-DDB sensor, we transfected human skin fibroblasts with a construct that drives the expression of DDB2 conjugated to enhanced green-fluorescent protein (EGFP). The DDB2-EGFP fusion, located in the nucleus, was subjected to protein dynamics studies by FRAP, which is a powerful real-time method to monitor the *in situ* mobility of DNA repair subunits in living cells [29]–[31]. A nuclear area (∼4 μm^2^) of human skin fibroblasts expressing low levels of DDB2-EGFP is bleached with a 488-nm wavelength laser that does not produce DNA damage. Subsequently, the replacement of bleached DDB2 molecules with non-bleached counterparts, diffusing from surrounding nuclear regions, leads to a progressive recovery of fluorescence in the target area (**Figure 1A**). Besides the diffusion properties of each protein, the rate by which this fluorescence intensity returns to pre-bleach levels depends on possible protein associations with DNA, chromatin fibers or nuclear scaffolds. As the EGFP tag itself undergoes minimal such interactions [32], the fluorescence signal associated with the DDB2-EGFP fusion recovers much slower than that of EGFP alone (**Figure 1B**).

Although UV-DDB is thought to be required only for the repair of UV lesions [24], [25], [30], previous biochemical assays showed that it also binds to other forms of DNA damage, including base adducts induced by the antitumor agent cisplatin [33]. To validate this FRAP assay as a probe of UV-DDB interactions with chemically induced DNA damage, fibroblasts transfected with DDB2-EGFP were pre-incubated with 5 μM cisplatin representing a non-cytotoxic drug concentration [34]. The bleached area ultimately regained the initial fluorescence intensity within ∼25 s, reflecting rapid movements of EGFP-tagged UV-DDB complexes within the nuclei. However, cisplatin exposure led to a transient delay in fluorescence recovery (**Figure 1C**), indicating that the nuclear trafficking of UV-DDB is slowed down by interactions with cisplatin-DNA adducts. From these findings, we concluded that FRAP provides a suitable method to monitor the interaction of UV-DDB with chemically damaged chromatin.

### Reduced UV-DDB trafficking by low-dose formaldehyde

**Figure 2:**
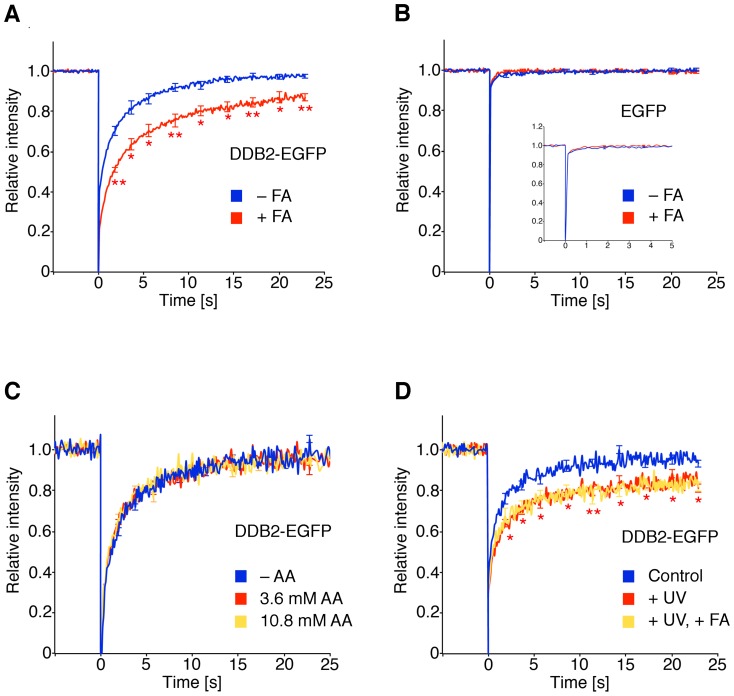
Delayed nuclear trafficking of UV-DDB. (**A**) FRAP analysis in human fibroblasts. Cells were transfected with DDB2-EGFP, incubated for 18 h with 75 μM formaldehyde (FA) and analyzed by FRAP (N = 50; error bars, S.E.M.). The fluorescence recovery curves were compared to those of untreated controls (*p<0.05; **p<0.01). (**B**) FRAP studies (N = 15; error bars, S.E.M.) demonstrating that EGFP movements are not affected by formaldehyde (18 h, 75 μM). (**C**) FRAP analysis with analogous acetaldehyde (AA) treatments (N = 50). (**D**) Combined formaldehyde and UV treatment. Transfected human fibroblasts were exposed to 75 μM formaldehyde for 18 h, UV-irradiated (30 J/m^2^) and subjected to FRAP analysis (N = 30). The asterisks indicate significant differences between UV-damaged fibroblasts and untreated controls (*p<0.05; **p<0.01).

Next, human fibroblasts expressing DDB2-EGFP were exposed for 18 h to a formaldehyde concentration (75 μM) that was selected to remain below the cytotoxic range but clearly above the reported physiological blood content, ranging between 13 and 20 μM [35]. The extended-time treatment was carried out to reflect the long-term exposure to formaldehyde in the population. Compared to the untreated control cells, the fluorescence recovery of DDB2-EGFP was retarded in formaldehyde-treated fibroblasts (**Figure 2A**), demonstrating a restrained movement of UV-DDB. Conversely, the fast fluorescence recovery observed with the EGFP moiety remained unchanged after formaldehyde treatment (**Figure 2B**), implying that interactions between DDB2 itself and formaldehyde-damaged chromatin are responsible for the reduced mobility. The same experiment was repeated with acetaldehyde at concentrations (≥3.6 mM) that have been shown to induce at least as many DPXs as 125 μM formaldehyde [36]. However, even at the highest dose, acetaldehyde did not interfere with the nuclear trafficking of UV-DDB (**Figure 2C**), pointing to a specific DDB2 response to formaldehyde damage. The distinct chemistry of the covalent linkage, the varying DNA structure at the site of the linkage or the particular pattern of covalently linked proteins [37] might explain this different effect of the two crosslinkers.

Because UV-DDB is implicated in DNA repair of UV lesions, we also monitored its mobility in cells transfected with DDB2-EGFP and then exposed to UV radiation, or in cells subjected to a dual treatment with both formaldehyde and UV light. The joint exposure did not further slow down the DDB2 movements compared to UV radiation alone (**Figure 2D**), and this lack of additive interaction suggests that the binding of UV-DDB to formaldehyde lesions and UV photoproducts is mutually exclusive. This view is supported below by the observation that low-dose formaldehyde hinders UV-DDB from recognizing the UV lesions.

### Non-covalent binding to formaldehyde-damaged chromatin

**Figure 3:**
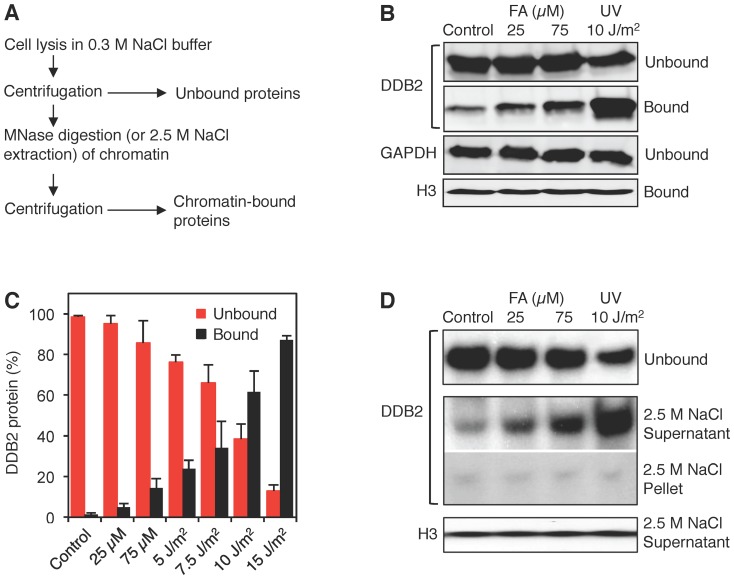
Association of UV-DDB with damaged chromatin. (**A**) Flow diagram illustrating how chromatin was dissected to monitor the binding of UV-DDB. Unbound proteins were released by salt (0.3 M NaCl) extraction and the remaining chromatin was solubilized by MNase digestion. (**B**) Western blot visualization of the chromatin partitioning of UV-DDB using antibodies against DDB2. GAPDH (glyceraldehyde 3-phosphate dehydrogenase), marker of unbound proteins; histone H3, marker of chromatin. Human fibroblasts were exposed for 18 h to formaldehyde or UV-irradiated at the indicated doses. (**C**) Quantification of three independent binding assays demonstrating the differential interaction of DDB2 with formaldehyde- and UV-damaged chromatin (error bars, S.D.). (**D**) Release of chromatin-bound DDB2 and histone H3 by high-salt extraction. After incubation with 0.3 M NaCl buffer, the chromatin was dissolved with 2.5 M NaCl, thus liberating non-covalently bound chromatin proteins.

The FRAP experiments revealed that UV-DDB remains attached to formaldehyde-damaged chromatin, such that its nuclear movement is inhibited. To confirm this finding, chromatin associations were tested biochemically as outlined in **Figure 3A**. First, free UV-DDB not bound to chromatin was removed by salt (0.3 M NaCl) extraction. Second, the resulting nucleoprotein complexes were solubilized by MNase, which liberates chromatin by cleavage into short nucleosomal repeats [38]. The fractions of free proteins (released by 0.3 M salt) and chromatin-bound proteins (released by MNase digestion) were analyzed using antibodies against endogenous DDB2 and different markers to compare their distribution following each treatment.

Upon exposure of human fibroblasts to increasing doses of UV light, a growing proportion of the cellular DDB2 pool translocated to chromatin and essentially all of this chromatin-bound DDB2 is released by MNase digestion (**Figure 3B and 3C**). In contrast, 18-h formaldehyde exposures induced a comparably weak binding of DDB2 to damaged chromatin. Even a 75-μM formaldehyde treatment, which in the FRAP experiments (**Figure 2**) reduced the protein mobility as much as a UV dose of 30 J/m^2^, resulted in considerably less chromatin association of DDB2. Importantly, the residual DDB2 that remained in chromatin after the 0.3 M salt treatment was completely released, in the absence of MNase digestion, by high salt (2.5 M NaCl) extraction (**Figure 3D**). These biochemical analyses demonstrate, therefore, that the association of UV-DDB, of which DDB2 constitutes the DNA-binding subunit, with formaldehyde-damaged chromatin occurs through transient non-covalent interactions. It is important to note that standard protocols that employ this reactant as a biochemical crosslinking agent, for example in chromatin immune-precipitation studies, use nearly 5,000-fold higher concentrations.

### Inhibition of XPC trafficking by formaldehyde

**Figure 4:**
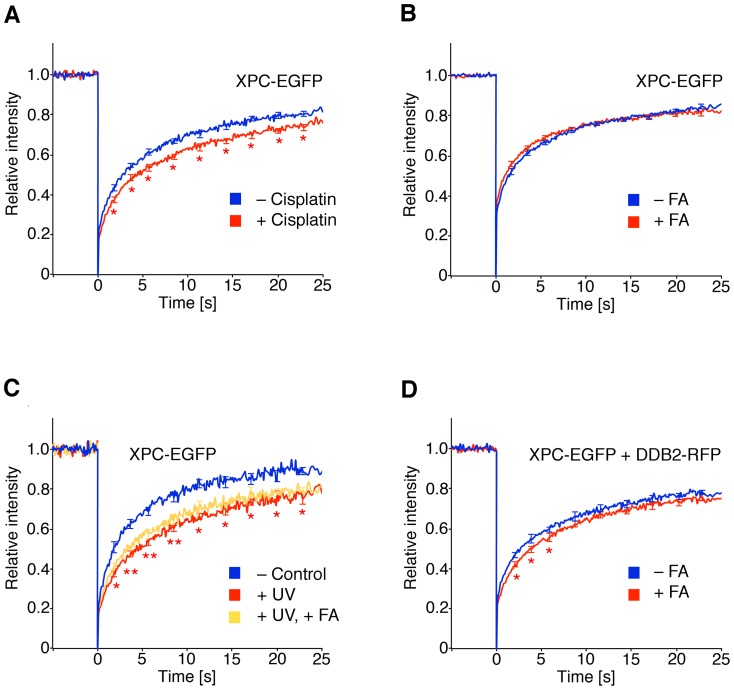
Indirect formaldehyde-induced reduction of XPC mobility. (**A**) Protein dynamics studies of XPC in the nuclei of human fibroblasts. Cells were transfected with the XPC-EGFP construct, incubated with 5 μM cisplatin and subjected to FRAP analysis (N = 30; error bars, S.E.M.). The resulting fluorescence recovery curves were compared to those of untreated controls (*p<0.05). (**B**) FRAP studies (N = 50) demonstrating that the fluorescence recovery curves of XPC-EGFP are not affected by an 18-h formaldehyde treatment (75 μM). (**C**) FRAP analysis of transfected fibroblasts demonstrating that the 18-h formaldehyde treatment (75 μM) does not further reduce the delayed XPC-EGFP trafficking in UV-irradiated cells (30 J/m^2^; N = 50). (**D**) Combined formaldehyde treatment and DDB2 overexpression. The transfected fibroblasts were exposed to 75 μM formaldehyde for 18 h and subjected to FRAP analysis. The presence of DDB2-RFP resulted in a slightly delayed fluorescence recovery curve of XPC-EGFP upon formaldehyde exposure (N = 30). Asterisks, significant differences between formaldehyde-treated and untreated fibroblasts (*p<0.05).

We next tested the movement of XPC, a major interaction partner of UV-DDB [24], [25], [39]. Unlike other NER factors, XPC displays a constitutive binding to native DNA that retards its nuclear dynamics, thus leading to an incomplete fluorescence recovery even in undamaged cells [31]. Again, FRAP experiments were performed on nuclear areas of ∼4 μm^2^ in human skin fibroblasts expressing low levels of XPC-EGFP. The validity of this approach for monitoring the binding of XPC to chemically damaged DNA is demonstrated by a 5-μM cisplatin treatment, whereby the mobility of XPC-EGFP is reduced (**Figure 4A**) reflecting its ability to recognize cisplatin-damaged DNA. Unlike the DDB2 response, however, the dynamics of the XPC complex is not influenced by 18-h formaldehyde (75 μM) treatments either alone (**Figure 4B**) or in combination with UV light (**Figure 4C**). A reduction of XPC mobility is nevertheless detected in cells that, in addition to endogenous DDB2, express ectopic DDB2 conjugated to red-fluorescent protein (DDB2-RFP; **Figure 4D**). This finding indicates that the XPC complex is indirectly immobilized by formaldehyde damage through its association with UV-DDB. Thus, in view of its low constitutive nuclear mobility, it is necessary to raise the level of the DDB2 interaction partner to visualize a sequestration of XPC protein in low-dose formaldehyde-damaged chromatin.

### Impaired recognition of UV lesions

The reduced nuclear trafficking of UV-DDB and XPC indicates that formaldehyde may hinder recognition of their common targets. To test this hypothesis, we examined the UV damage recognition function of UV-DDB and XPC complexes in living fibroblasts. First, cells expressing low levels of the DDB2-EGFP construct were UV-irradiated through the pores of polycarbonate filters, thus focalizing DNA damage to narrow nuclear spots. After a 15-min recovery, DNA damage recognition was demonstrated by recording the co-localization of DDB2, detected by fluorescence measurements, and UV lesions detected by immunocytochemistry.

**Figure 5:**
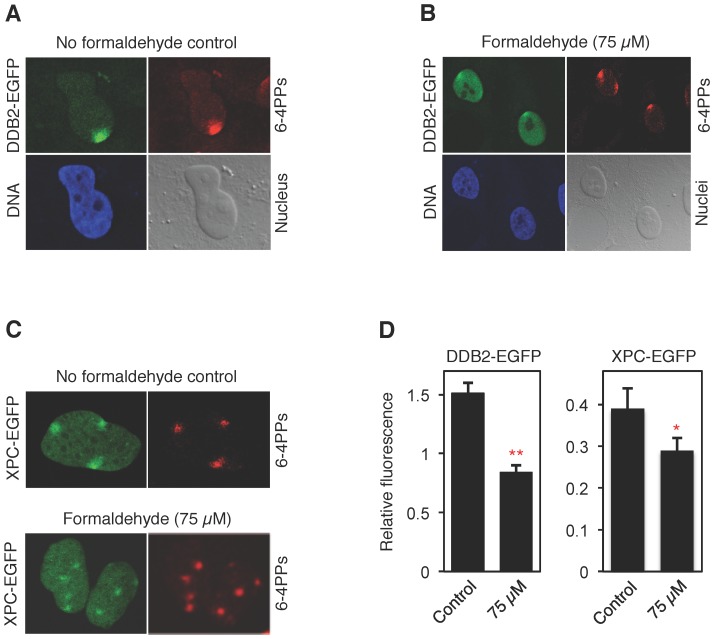
Accumulation of damage recognition factors on UV lesions. (**A**) Representative image illustrating the redistribution of DDB2 to UV lesion sites visualized with antibodies against (6-4) photoproducts. Human fibroblasts transfected with DDB2-EGFP were UV-irradiated through the pores of polycarbonate filters and fixed 15 min after treatment. DNA is evidenced by the Hoechst reagent and the nuclei are shown with contrast images. (**B**) Representative cells demonstrating the defective translocation of DDB2-EGFP from undamaged nuclear areas to UV lesions after 18-h incubations with 75 μM formaldehyde. (**C**) Representative images illustrating that the 75-μM formaldehyde treatment impedes the redistribution of XPC-EGFP to UV lesions. (**D**) Reduced fluorescence intensity at UV lesion spots over the surrounding background in cells exposed for 18 h to 75 μM formaldehyde (N = 54; error bars, S.D.; *p<0.05, **p<0.01).

**Figure 5A** shows that the UV-dependent nuclear redistribution of DDB2-EGFP induces bright green spots accompanied by a reduced overall nuclear fluorescence. This pattern reflects an efficient accumulation of UV-DDB at damaged sites with concomitant depletion of the protein from undamaged nuclear regions containing no lesions. In 75-μM formaldehyde-treated cells, UV-DDB still relocated to UV-irradiated sites, but less efficiently, resulting in weaker spots of green fluorescence signals over the surrounding nuclear background (**Figure 5B**). A similarly reduced relocation to UV lesion spots was observed in cells transfected with XPC-EGFP and exposed to formaldehyde (**Figure 5C**). The quantitative comparison of three experiments confirmed that formaldehyde impairs the UV-dependent redistribution of both UV-DDB and XPC, thus inhibiting their translocation from undamaged nuclear areas to UV-irradiated sites. In each case, the ratio of fluorescence intensity at UV lesion spots against the surrounding background was reduced by low-dose formaldehyde (**Figure 5D**).

### Differential effects on BER enzymes

**Figure 6:**
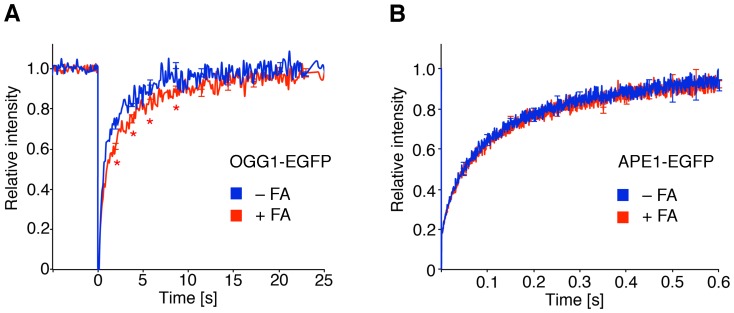
Formaldehyde-induced damage delays the nuclear trafficking of a DNA glycosylase. (**A**) Nuclear dynamics of OGG1, the DNA glycosylase that removes 8-oxo-dG, in human fibroblasts. Cells were transfected with the OGG1-EGFP construct, incubated for 18 h with 75 μM formaldehyde and subjected to FRAP analysis (N = 50; error bars, S.E.M.). The resulting fluorescence recovery curves were compared to those of untreated controls (*p<0.05). (**B**) FRAP studies (N = 50) demonstrating that the extremely fast movements of the APE1-EGFP fusion are not affected by the 75-μM formaldehyde treatment.

Another permanent trigger of mutagenesis are oxidative base lesions such as 8-oxo-dG [3]–[5]. Therefore, we tested whether formaldehyde affects the nuclear dynamics of 8-oxo-dG-DNA glycosylase 1 (OGG1), which initiates BER by recognizing and removing 8-oxo-dG from DNA leaving apurinic sites. The FRAP experiments of **Figure 6A** revealed that, in fibroblasts transfected with OGG1-EGFP, the fluorescence recovery is delayed upon incubation with 75 μM formaldehyde, indicating that the constitutive movement of this DNA glycosylase is disturbed by low-dose formaldehyde. Then, we probed the nuclear dynamics of an immediately downstream enzyme in the BER pathway, i.e., apurinic/apyrimidinic endonuclease 1 (APE1). This follow-up BER subunit displayed much faster movements in FRAP assays that were refractory to the 75-μM formaldehyde treatment (**Figure 6B**).

### Inhibition of excision activity

**Figure 7:**
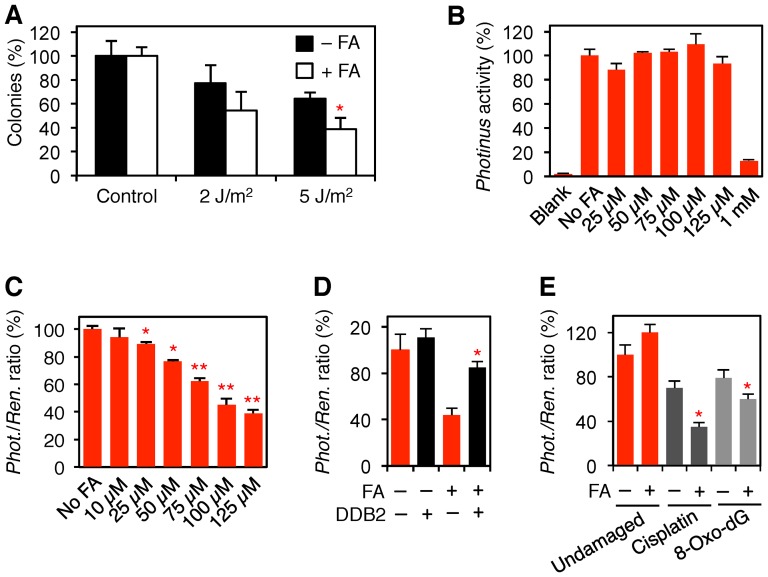
Inhibition of NER and BER activity by low-dose formaldehyde. (**A**) Colony-forming assay demonstrating that human fibroblasts exposed to formaldehyde (75 μM) are more sensitive to killing by UV radiation (2 and 5 J/m^2^) than the respective untreated controls (error bars, S.D.; n = 3, each measurement in triplicate). The asterisk (*p<0.05) denotes the significantly reduced colony formation ability. (**B**) Expression of *Photinus* luciferase in cells containing undamaged pGL3 and exposed (18 h) to formaldehyde. All values (N = 3; error bars, S.D.) are shown as a percentage of luciferase activity in untreated fibroblasts. Blank, untransfected cells. (**C**) Host-cell reactivation of UV-irradiated pGL3 reflecting NER activity in cells exposed (18 h) to formaldehyde. Values (N = 10; error bars, S.D.) are shown as a percentage of the *Photinus*/*Renilla* ratio in control cells. Asterisks, significant differences from the control (*p<0.05, **p<0.01). (**D**) Partial restoration of host-cell reactivation in 100-μM formaldehyde-treated cells overexpressing DDB2-EGFP (N = 5). Asterisk, significantly (*p<0.05) higher NER activity then controls without DDB2-EGFP. (**E**) Inhibition of host-cell reactivation of pGL3 containing cisplatin adducts or 8-oxo-dG lesions (N = 5). The asterisks (*p<0.05) denote significantly reduced DNA repair activity in formaldehyde-treated (75 μM, 18 h) cells.

Consistent with the compromised mobility of repair factors in the chromatin context, the low-dose (75 μM) formaldehyde treatment increased the sensitivity of human fibroblasts to the cytotoxic effect of UV light (**Figure 7A**). Next, the fibroblasts were transfected with a reporter vector (pGL3), carrying the *Photinus* luciferase sequence, to determine the consequence of low-dose formaldehyde exposures on transcription and translation. The measurement of reporter luciferase activity in cell lysates showed that formaldehyde at concentrations of up to 125 μM exerted no inhibition on RNA or protein (luciferase) synthesis (**Figure 7B**). However, this reporter expression was suppressed by increasing the formaldehyde concentration to 1 mM, which leads to overt cytotoxicity.

Next, to monitor the NER pathway, pGL3 reporter vectors coding for the *Photinus* luciferase were UV-irradiated and introduced into fibroblasts together with an undamaged control coding for *Renilla* luciferase. DNA repair efficiency was assessed by measuring the *Photinus* luciferase activity in cell lysates, followed by normalization against the *Renilla* control. In this dual reporter assay, reactivation of UV-irradiated pGL3 is dependent on the ability of the NER system to remove UV lesions. The resulting *Photinus*/*Renilla* luciferase ratios revealed that NER activity is significantly reduced by formaldehyde exposure at 25-μM or higher (**Figure 7C**). This low-dose formaldehyde effect provides a proof for DNA repair inhibition as it reflects diminished reactivation of the UV-irradiated template rather than reduced transcription or translation.

The findings of **Figure 5** indicated that the inhibition of DNA repair is caused by an impaired nuclear trafficking of UV-DDB, resulting in reduced recognition of UV lesions during the NER process. This view was confirmed by the observation that NER inhibition could be reversed by co-transfection of the dual reporter system with a DDB2-EGFP expression vector (**Figure 7D**). Thus, by raising the cellular DDB2 level in formaldehyde-treated cells, it was possible to restore the level of global-genome NER activity to that found in untreated cells. That the defective recognition function also jeopardizes the excision of chemically induced DNA damage is supported by host-cell reactivation assays where the pGL3 *Photinus* vector was damaged by cisplatin. Indeed, as a consequence of the inhibited removal of cisplatin-DNA adducts, expression of the cisplatin-damaged reporter was reduced by formaldehyde (**Figure 7E**). Finally, the induction of oxidative lesions, instead of UV or cisplatin adducts, in the same reporter vector demonstrated that also the hindrance of OGG1 mobility, observed in **Figure 6A**, translates to a significantly reduced BER efficiency as measured in host-cell reactivation assays (**Figure 7D**).

**Figure 8:**
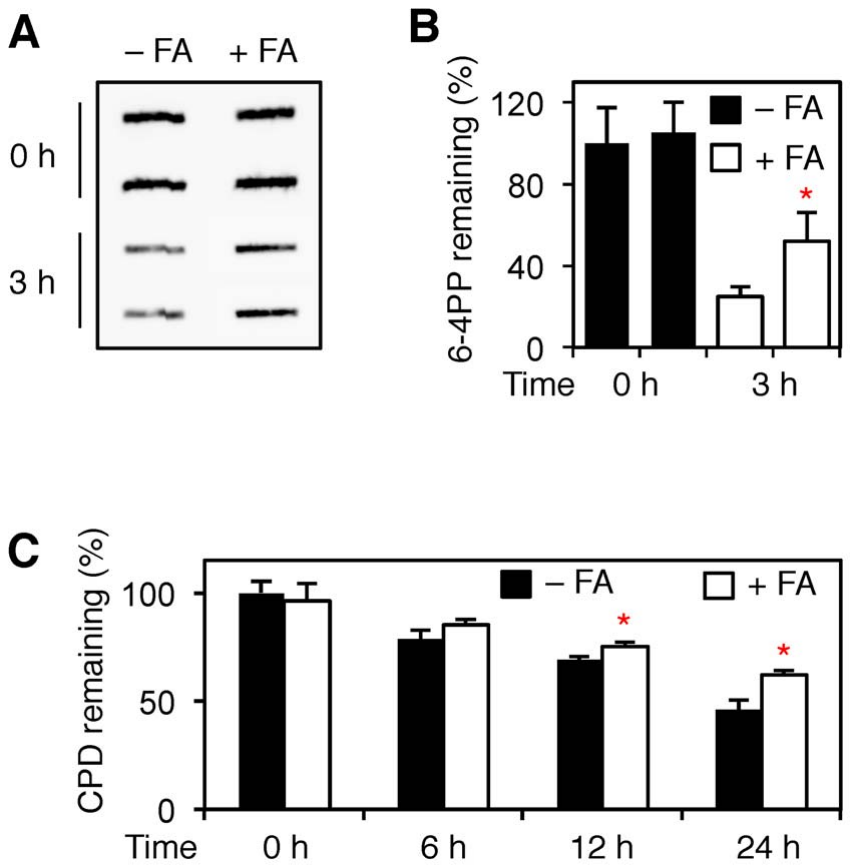
Inhibition of UV lesion removal from chromatin. (**A**) Untreated or formaldehyde-treated cells (75 μM, 18 h) were exposed to UV light (10 J/m^2^) and collected immediately after irradiation or following 3-h repair incubations. Genomic DNA was isolated and analyzed for UV lesions using antibodies against 6-4PPs. (**B**) Quantification of three independent experiments demonstrating that 6-4PP excision is diminished by 75-μM formaldehyde exposure (error bar, S.D.). The asterisk (*p<0.05) denotes significantly reduced excision in formaldehyde-treated cells compared to the untreated control. (**C**) Quantification of three independent experiments demonstrating that CPD excision is also inhibited. The asterisks (*p<0.05) denote the significantly reduced excision in 75-μM formaldehyde-treated cells compared to untreated controls.

To confirm that an inhibition of DNA repair activity occurs in the chromosomal context, human fibroblasts were UV-irradiated after a 75-μM pretreatment with formaldehyde. The excision of UV lesions from genomic DNA was monitored by a slot-blot immunoassay taking advantage of antibodies against (6-4) pyrimidine-pyrimidone photoproducts (6-4PPs; **Figure 8A**) or cyclobutane pyrimidine dimers (CPDs). Among the UV lesions induced by sunlight, 6-4PPs are the ones removed most rapidly from human cells [40]. In fact, only ∼25% of the initial amount of these 6-4PPs remained in the DNA of untreated control fibroblasts after a repair time of 3 h. In contrast, formaldehyde-exposed cells displayed a slower excision activity with only ∼50% photoproduct repair during the same 3-h incubation period (**Figure 8B**). Similarly, the low-dose formaldehyde treatment slowed down the excision of CPDs in human fibroblasts (**Figure 8C**).

## Discussion

The human skin is increasingly subjected to UV damage due to growing leisure times, the popularity of outdoor activities, frequent traveling to tropical areas and the use of artificial irradiation devices. Concomitantly, the DNA of the skin or mucous membranes is constantly attacked by exogenous or endogenous genotoxins. If not promptly excised by DNA repair machines, the resulting base lesions give rise to mutations, which in turn may cause cancer. However, DNA repair systems targeting such mutagenic lesions are prone to modulation by skin- or mucous membrane-penetrating xenobiotics. This is the first study that shows a direct effect of low-dose formaldehyde on the assembly of NER complexes. A previous report already suggested that formaldehyde delays DNA repair of UV lesions [23] but this earlier study was based on indirect measurements whereby transiently appearing single-stranded DNA breaks were taken as circumstantial evidence for ongoing excision repair. Another preceding report [22] revealed an inhibitory effect of formaldehyde on unscheduled DNA synthesis, reflecting DNA repair activity, but at considerably higher concentrations of > 100 μM.

One possible scenario to explain our findings is that formaldehyde-induced DPXs are themselves NER substrates that compete with UV lesions for being repaired. The processing of DPXs is not completely understood but recent studies concluded that the NER pathway plays only a marginal role in the removal of DPXs induced by low-dose formaldehyde [41]. Thus, the delayed NER activity is unlikely to result from direct substrate competition between DPXs and UV lesions. As an alternative mechanism, we hypothesized that the formation of DPXs, mainly histone-DNA crosslinks, may slow down the molecular search for UV lesions by DNA damage sensors. It is believed that site-specific DNA-binding proteins, including DNA repair factors, locate their targets among the vast excess of non-target DNA by facilitated diffusion. This search process reduces the dimensionality of protein movements by “sliding” or “hopping” along DNA filaments [42]. In either case, the presence of covalently trapped histones or other chromatin proteins along the DNA path may interrupt such an effective search mode by facilitated diffusion, thereby restricting the rate by which DNA lesions are detected and channeled into DNA repair. This hypothesis was tested by *in situ* protein dynamics, thus demonstrating that formaldehyde slows down the recognition of DNA damage by UV-DDB and XPC. That this reduced damage recognition efficiency translates to slower repair has been confirmed by two different tests. In a host-cell reactivation assay, NER activity was inhibited by formaldehyde with a 50% reduction at concentrations of 75-100 μM. A NER inhibition was similarly observed by monitoring the removal of 6-4PPs and CPDs from the chromosomal DNA of human cells exposed to 75 μM formaldehyde.

Mechanistically, we found that the observed sequestration of UV-DDB in formaldehyde-damaged chromatin results from transient, non-covalent interactions that delay its movements during the search for DNA damage. Through protein-protein associations, UV-DDB bound to formaldehyde-damaged chromatin also impedes the function of the XPC complex, such that not only the excision of UV lesions but also the repair of chemically induced DNA adducts is diminished. We also found that the mobility of a representative DNA glycosylase is perturbed by low-dose formaldehyde and that the ensuing BER pathway is inhibited. Further dynamic chromatin transactions may be disturbed by a similar mechanism. For example, Rager et al. [9] recently reported that formaldehyde perturbs gene expression in the nasal epithelium.

In conclusion, the compromised DNA repair efficiency, reported in this study, raises the possibility that frequent exposures of the skin or mucous membranes to formaldehyde represents an unexpected additional risk factor for cutaneous cancer in combination with UV radiation or chemical carcinogens. In a broader perspective, the inhibition of DNA excision repair activity by formaldehyde implies that the carcinogenic endpoints of this highly reactive aldehyde may result, to a great extent, from the accumulation of DNA adducts and other mutagenic base lesions induced by constitutively occurring environmental carcinogens or endogenous genotoxic metabolites. In view of this adjuvant effect, we expect that a reduction of formaldehyde exposure in the general population would substantially reduce the overall cancer incidence.
